# The Paradox of Nutrition-Related Diseases in the Arab Countries: The Need for Action

**DOI:** 10.3390/ijerph8093637

**Published:** 2011-09-08

**Authors:** Abdulrahman O. Musaiger, Abdelmonem S. Hassan, Omar Obeid

**Affiliations:** 1 Nutrition and Health Studies Unit, Deanship of Scientific Research, University of Bahrain, Sakhair, Zallaq Street, Bahrain; 2 Arab Center for Nutrition, Street 1535, Muharraq, Bahrain; 3 Department of Health Sciences, Qatar University, University Avenue, Qatar; E-Mail: ahassan@qu.edu.qa; 4 Department of Nutrition and Food Science, Faculty of Agricultural and Food Sciences, American University of Beirut, Beirut, Bliss Street, Lebanon; E-Mail: Omar.obeid@aub.edu.lb

**Keywords:** Arab countries, nutrition problems, undernutrition, diet-related chronic non-communicable disease

## Abstract

The aim of this review was to highlight the current situation of nutrition-related diseases in the Arab countries, and factors associated with prevalence of these diseases. PubMed and Google Scholar were searched for data relating to such nutrition-related diseases published between January 1990 and May 2011. The picture of nutritional status in the Arab countries has changed drastically over the past 30 years as a result of changes in the social and economic situation. Two contrasting nutrition-related diseases exist, those associated with inadequate intake of nutrients and unhealthy dietary habits such as growth retardation among young children and micronutrient deficiencies; and those associated with changes in lifestyle such as cardiovascular disease, cancer, osteoporosis, diabetes and obesity (diet-related non-communicable diseases). Factors contributing to nutritional problems vary from country to country, depending on socio-economic status. In general, unsound dietary habits, poor sanitation, poverty, ignorance and lack of access to safe water and health services are mainly responsible for under-nutrition. Changes in lifestyle and dietary habits as well as inactivity are associated with the occurrence of diet-related non-communicable diseases. Programs to prevent and control nutrition-related diseases are insufficient and ineffective, due mainly to a focus on curative care at the expense of preventive health care services, lack of epidemiological studies, lack of nutritional surveillance, inadequate nutrition information and lack of assessment of the cost-effectiveness of nutrition intervention programs.

## 1. Introduction

Nutrition-related diseases manifested by under-nutrition and over-nutrition are leading contributors to the disease burden in the Arab countries. Under-nutrition remains one of the most serious health problems among preschool children and the single main contributor to child mortality in many low and middle income Arab countries. A significant proportion of children are undernourished, and over a third of the population in this region suffers from micronutrient deficiencies [1]. On the other hand, diet-related non-communicable diseases such as obesity, heart disease, diabetes and cancer have become the main public health problems among adults in almost all the Arab countries. Both under-nutrition and over-nutrition have a great negative impact on social, economic and public health care systems. Therefore, intervention programmes to control these nutritional problems should consider political, food security, economic, social, agriculture and environmental factors in order to develop multi-sectoral programmes [2]. This review discusses the magnitude of the nutrition-related diseases in the Arab countries, their causes, and the current programmes to overcome these diseases.

## 2. Methods

The search was restricted to articles and reports on nutrition problems and diet-related diseases published in English from January 1990 to May 2011. We performed our search using PubMed and Google Scholar, together with our own search on reports published by official and international organizations in the region. The search covered only the Arab world, which refers to the Arabic speaking countries stretching from the Atlantic Ocean in the west to the Arabian Sea in the east, and from the Mediterranean Sea in the north to the Horn of Africa and Indian Ocean in the southeast.

Several keywords were used in the Search. These included: nutrition, diet, food intake, diet-related non-communicable diseases, food security, vitamins deficiency, anemia, iodine deficiency, low birth weight and malnutrition for each Arab country. From this initial search, we identified more than 5,000 original and review papers, as well as reports. The titles and abstracts of these papers and reports were then reviewed to screen for publications that potentially addressed the nutrition situation in the Arab Region. At this stage we included the following:

1. For prevalence of nutritional problems and diet-related non-communicable diseases, only national-based data were included. This was done for overweight and obesity. As for other diseases, the data by World Health Organization and UNICEF were used, as they represented national data obtained from official bodies in Arab countries. Therefore, studies on prevalence of diseases in town, rural, urban, regional or hospital-based were excluded (most exclusions were in this category).
2. For the factors associated with nutritional problems, one to three examples for each factor were included as possible. Selection of the factor was based on year of publication, sample size and the variation of countries. Therefore, many publications were excluded for this purpose.
3. Studies on purely genetic or biological nature and those applies on animals were all excluded, as the current paper is an epidemiological review on nutrition in the Arab countries.
4. All papers published on Arab people living outside the Arab countries were also excluded.

After this screening we indentified 430 articles that fitted our selection criteria. After a subsequent screening of these articles, we included 125 papers in this review.

## 3. Findings and Discussion

### 3.1. Undernutrition and Micronutrient Deficiencies

Under-nutrition and micronutrient deficiencies in the Arab Region can manifest themselves in low growth rate in children low birth weight, deficiencies in iron, iodine and vitamins A and D.

#### 3.1.1. Low Birth Weight

Low birth weight (LBW) is used as an indicator for the assessment of the social and health status of a community. The prevalence of LBW (<2.5 kg) in the Arab countries, ranges from 6% in Lebanon to 32% in Yemen [3]. It is highly believed that LBW is one of the most important factors for infant mortality in the Arab Region. In Yemen, for example, LBW is the third leading cause of death in children [4]. Use of a multivariate model indicated that low birth weight Egyptian children were about three times more likely to die in infancy than normal weight children (odd ratio: 2.89, 95% CI: 2.33–3.58) [5]. Studies on factors associated with low birth weight in the Region are few and limited. These suggest that female sex, low socioeconomic status, older mothers and smaller interval between pregnancies are main risk factors linked to low birth weight. Gestational iron deficiency anemia may also play an important role in the prevalence of low birth weight in this Region [2]. In the United Arab Emirates, using a multiple logistic regression model, the main risk factors for low birth weight were: gestational age, previous LBW delivery, maternal BMI <19, multiple births and absence of housework-help [6]. Malnutrition among mothers is another important factor in some countries such as Iraq, Djibouti, Somalia, Sudan and Yemen [2,3]. In poor countries, malaria is another considerable factor for LBW. In Yemen, it was reported that malaria was significantly associated with LBW (odds ratio 5.7, 95% CI 1.7–18.5 [7]. In Sudan, maternal malaria was the most important factor for LBW [8].

Data on the effects of maternal weight gain on infant birth weight in Arab countries are lacking. It was shown that women with lower first trimester BMI (<25) had infants of lower weight than women of higher BMI (≥25). Women with lower gain (<35 lbs) delivered smaller infants than women with higher gain (≤35 lbs). Women with higher BMI and higher gain delivered the largest infants (P < 0.0015) [9].

#### 3.1.2. Under-Nutrition

Undernutrition, which can be categorized as being underweight (low weight for age), stunting (low height for age) and wasting (low weight for height), is common among preschool children (<5 years) in all Arab countries. Stunting is the most common type of undernutrition in these countries, followed by underweight and wasting. It has been estimated that the prevalence of stunting among children under five years ranges from 8% in Qatar to 53% in Yemen, while the prevalence of underweight ranges from 3% to 61% and the proportions for wasting ranges from 3% to 16% [3,10]. In general, poor countries like Djibouti, Mauritania, Somalia, Sudan and Yemen have the highest prevalence of under-nutrition among under five years children, compared to other countries. For example, the prevalence of moderate cases of underweight among children below five years ranged between 28.9% to 45.6% in poor Arab countries, compared to 3.9% to 17.8% in other countries (Table 1).

Several factors contribute to undernutrition among preschool children in the Region. Among poorer countries, such as Djibouti, Somalia, Sudan and Yemen, poor sanitation, unhygienic environment, lack of access to safe clean water, parasite infections, poverty, illiteracy, low availability of foods and the lack of health services, are the main factors responsible for the high prevalence of undernutrition. Recurrent diarrhea is still an important factor contributing to undernutrition in both poor and middle-income countries, (such as Egypt, Syria, Morocco, Tunisia, Jordan, Algeria and Libya). In higher income countries, such as the Arab Gulf countries, factors contributing to undernutrition among this age group are chiefly unhealthy dietary habits and lack of nutritional awareness [3,12].

A review on disparities in child health in 18 Arab countries indicated that 13 countries had higher rates of stunting for male than female children. Palestine, however, showed a clear female disadvantage, but with regard to wasting; there was a clear male disadvantage. Only Jordan, Sudan and Tunisia had significantly higher rates of female child wasting. Female advantage in child nutrition could be attributed to biologically higher susceptibility of boys to diseases such as diarrhea and acute respiratory infection, and their consequent failure to thrive [13].

Underweight has also been reported among school children (6–11 years) and adolescents (12–18 years) in many Arab countries. Studies in Egypt, Lebanon, Tunisia, Jordan, Yemen and Arab Gulf countries showed that the prevalence of underweight ranged from 10% to 35% among school children and 5% to 25% among adolescents. In spite that most of these studies were not national baselines, they give a good indicator that a high percentage of underweight exists in these age groups in the Region. Unhealthy dietary habits such as skipping breakfast, low intake of nutritious foods (such as milk, fruits and vegetables), high intake of empty calories foods (such as soft drinks and some sweets) and lack of nutritional knowledge are the main factors for the high proportion of underweight among school children and adolescents in the majority of Arab countries[14,15].

The result of correlation coefficients suggest that the child malnutrition and mortality in most of Arab countries have declined in the last three decades. These declines are mainly as a result of marked progress in health care and services, as well as the general improvement in socio-economic brought by the oil wealth, which have done much to lower child mortality, particularly in the Arab Gulf countries. Also the findings indicate that child malnutrition is sensitive responses to both health programmes and socioeconomic conditions such as income, unemployment and illiteracy [16].

#### 3.1.3. Anemia

On a nationwide basis, the main type of anemia is iron deficiency anemia (IDA). About 50% of anemia cases are generally traced back to iron deficiency, although this proportion varies between different population groups and regions. When compared to other developing countries, anemia appears to be a moderate public health problem (20–39.9%) [17]. Studies have shown the prevalence of anemia in countries of the Arab Gulf to be 20–67% among preschoolers, 13–50% among school aged children and 23–54% in women of childbearing age [18]. Other studies in the Region have similarly shown the prevalence among pre-schoolers to be 17–70%, with it being lower in adolescents (14–42%) and around 11–40% in pregnant women [19]. Data presented in Table 2 indicate a very high prevalence of anemia among children 0–59 months in most Arab countries. Somalia (78%), Mauritania (73.8%), Sudan (70%) and Oman (60%) have the highest prevalence. As for women, the prevalence of anemia ranged from 22.3% in Lebanon to 46.1% in Egypt. The prevalence continue to be high among non-pregnant women aged 15–40 years (ranged from 12.3% to 53.7%).

Iron deficiency anemia was found to be the most common type of anemia in all Arab countries [20]. The causes of IDA have been reported as being associated with low intakes of total dietary iron, high consumption of non-heme compared to heme iron, low intake of foods capable of enhancing the absorption of iron (such as fruit and vegetables containing Vitamin C), poor iron absorption due to iron-inhibiting factors such as tannins and phytates, unhealthy eating patterns, parasitic infections, early marriage and parity, and ethnic differences [18,19]. In Kuwait it was found that pregnant women with higher gestational age, short birth spacing (<2 years), not taking iron-folate tablets, not consuming fruit juice, consuming brown bread, tea and/or coffee were at higher risk for iron deficiency anemia [21]. In Lebanon it was showed that IDA, found to be quite common among Lebanese children 11–72 months (about 20.5%), and was associated with high levels of plasma lead, lack of iron supplementation and cultural dietary patterns [22]. The high consumption of tea may have a role in etiology of IDA, especially in poor Arab countries, where the intake of high biological iron is low. In Morocco, it was reported that tea inhibits iron availability among infants less than one year (infants during weaning), and in contrast, mint improves it. It was concluded to discourage tea drinking at early weaning and to replace them by mint infusion or promoting the intake of vitamin-rich fruit juice to counteract these inhibitory effects [23]. Low iron bioavailability from legume and cereal-based diets is also found to be a cause of iron deficiency anemia in children in rural Morocco [24]. Other general causes of anemia include heavy blood loss (as in menstruation), parasitic infections (such as hookworms, ascaris, and schistosomiasis), acute and chronic infections (e.g., malaria, cancer, tuberculosis, and HIV) and the presence of other micronutrient deficiencies, namely those associated with vitamins B^2^, B^6^, B^12^, A and the mineral copper [17]. Childhood, adolescence and pregnancy are three stages of the life cycle where IDA seems to be highly prevalent, mainly due to the increased iron needs of the growing and developing body.

#### 3.1.4. Vitamin A Deficiency

The term vitamin A deficiency (VAD) embraces all forms and degrees of deficiency including the most severe, in which the function and structure of the eye are affected, leading to blindness [25]. Vitamin A deficiency has been found to be less common in developed than in developing countries, especially among preschool and school-aged children and women of childbearing age, where malnutrition tends to be of greater concern. In general, vitamin A deficiency in the Arab Region prevails at mild to moderate levels. Commonly used indicators of Vitamin A deficiency, such as a serum retinol concentration of <20 μg/dL, xerophthalmia, night blindness and Bitot’s spots, serve as proxies for the detection of low vitamin A status among children 0–72 months of age. National surveys conducted from the year 1990–2000 in the Arab Region revealed an improvement in the patterns of both xerophthalmia and vitamin A deficiency. It was estimated that the prevalence of vitamin A deficiency among children 0–72 months, decreased from 32.6% in 1990 to 28% in 2000 [11]. Data presented in Table 2 showed that the prevalence of vitamin A deficiency among 0–72 months children was very high in Iraq (40%) and Sudan (35.8%), while the prevalence ranged from 13.7% in the UAE to 29.2% in Morocco.

Low intake of dietary vitamin A was reported in Egypt [26] and Sudan [27]. Fawzi *et al.* [27] sought to prospectively examine the relationship between dietary vitamin A intake and child growth in Sudan. They found that total dietary vitamin A intake was associated with height and weight attainment among children who were normally nourished at baseline after controlling for age, sex, morbidity, and socioeconomic variables. However, providing the same study population with vitamin A supplements did not have any considerable effects on either the rate of height or weight [28]. The association of vitamin A deficiency with other diseases such as anemia may complicated the problem. In Jordan, more than 50% of Bedouin children aged 6–66 months were at risk of vitamin A deficiency and children with this deficiency tended to have lower hemoglobin (10.0 g/dL *vs.* 10.95 g/dL, p < 0.001) and lower serum ferritin concentration (9.2 micro g/L *vs.* 16.3 micro g/L, P < 0.001) [29]. Thus, improving access to vitamin A rich foods rather than giving vitamin A supplements seems to be of greater effectiveness in improving the nutritional status of malnourished populations in which vitamin A deficiency is an area of concern.

#### 3.1.5. Vitamin D Deficiency

Hypovitaminosis D can result from low sunlight exposure in addition to an inadequate dietary intake of vitamin D. An adequate vitamin D and calcium intake is essential for optimal peak bone mass accretion during childhood and adolescence since it is a period of rapid skeletal growth. Nevertheless, information concerning vitamin D intake in the Region is scarce. There is a relatively high prevalence of hypovitaminosis D in Arab countries although these are sunny countries. In adults, it is reported that the prevalence of low bone mass in the Arab world is higher than in Western [30,31]. Average daily intakes of calcium and vitamin D among young healthy Lebanese 30–50 years of age were estimated to be 638 ± 281.2 and 100.6 ± 71.0 IU respectively, both of which are below the recommended daily intakes. In this population, vitamin D sources were equally provided from milk and dairy products (30.2%) meat and poultry (28%) and fish (25.6%) [32].

Bener *et al.* [33] conducted a cross-sectional study with the aim of determining the prevalence of vitamin D deficiency amongst 458 Qatari children (males and females) less than 16 years of age. The study revealed a high prevalence of vitamin D deficiency (68.8%); this was found to prevail mainly in the 11–16 years age group and was more common among girls. This was mainly related to low sunlight exposure (especially among girls), breast feeding for a duration of less than 6 months, low dietary vitamin D intake, family history of diabetes mellitus and low physical activity among this group. The prevalence and risk factors for vitamin D inadequacy amongst Lebanese osteoporotic women were investigated [34]. The population group consisted of 251 Lebanese postmenopausal osteoporotic women. The prevalence of vitamin D inadequacy deficiency (25-hydroxyvitamin D (25(OH)D) < 30 ng/mL) was 84·9%. Moreover, 25(OH)D, was negatively correlated with BMI and positively correlated with educational level, vitamin D supplement intake and self-reported general health. However, no significant correlation was found with age and no seasonal variation was observed.

Several studies raised the issue of traditional clothing of women in the Arab world, like wearing veil or those who covered whole body, as a factor for vitamin D deficiency [35–37]. No significant differences were noted between Jordanian women wearing different dress styles and prevalence of vitamin D deficiency [35]. In Tunisia, multiparity, menopause, wearing the veil, and calcium and vitamin D dietary intake were found to be associated with hypovitaminosis D (P < 0.05). However, when logistic regression was used, only multiparity and vitamin D dietary intake were independent predictive factors [36]. In Morocco, Allali *et al.* [37] found that Moroccan post menopausal women, wearing a traditional concealing clothing covering arms, legs and head increased the risk of osteoporosis (OR = 2.20, 95 CI 1.22–3.9).

Overall, these studies demonstrate that hypovitaminosis D is widespread among Arab countries and that the need to consider mandatory vitamin D food fortification or supplement use, particularly during the winter time and encouraging more active outdoor lifestyle, is critical in correcting this deficiency state.

#### 3.1.6. Iodine Deficiency Disorders

Iodine deficiency disorders (IDDs) prevail in many areas of the World and include a constellation of preventable nutritional disorders that have an impact on both social and economical status. These disorders, some being irreversible, include goiter (an enlargement of the thyroid gland), decreased fertility, cretinism (mental retardation) and growth impairment. According to the WHO criteria, iodine deficiency is identified when the median urinary iodine (UI) level is below 10 μg/dL or goiter prevalence is greater than 5% in school children [38]. Based on these criteria, the Arab countries have a high proportion of the total World goiter prevalence (TGP); in 1993, a prevalence rate of 22.9% for goiter was recorded for the Region [39]. This value has increased to 37.3% in 2003 portraying a 62.9% increase as compared to 1993 [40]. In 2003, the World Health Organization estimated that the proportion of school-age children (6–12 years) and the proportion of the general population with insufficient iodine intake based on urinary iodine levels in the Region was 55.4% and 54.1% respectively, placing it as the second most affected Region after Europe (59.9% and 56.9% respectively) [40]. The prevalence of IDD is considered mild in seven countries of the Region including Jordan, Lebanon, Libyan Arab Jamahiriya, Oman, Syria, the United Arab Emirates and Yemen, whereas it is found to be moderate in four countries that include Egypt, Morocco, Saudi Arabia and Sudan [25].

The prevalence of total goiter in Arab countries ranged from 5% to 70%. The countries most affected by total goiter were Iraq, Jordan, Lebanon, Morocco, Sudan, Syria and Yemen. The iodine deficiency status (IDD) in the Region ranged from mild to moderate, with the exception of Iraq, where the IDD status is severe most likely due to inadequate intake of dietary iodine, ingestion of goitrogens (food contain chemical inhibit iodine absorption) and habitation in Regions where the soil lacks iodine [25]. Lack or inadequate intake of fish may contributed to iodine-deficiency. In Morocco, fish consumption was negatively associated with goiter among school children in Atlas Mountains of Morocco. It was concluded that the effective programme to control the prevalence of goiter should include four main activities: encourage fish consumption, salt iodization, nutrition education [41]. However, several studies showed that the altitudes may play an important factor. In Saudi Arabia, the overall prevalence of goiter among school children 6–18 years was 24%. The prevalence was significantly higher (P < 0.000) in high altitude (27%, 95% CI, 24–30%) than in low altitude areas (13%, 95%, CI 8–18%) [42]. In Yemen, the prevalence of IDD among school children 6–12 years in mountain areas was 31% compared to 16.8% in lowland/coastal areas [43].

### 3.2. Over-Nutrition: Diet-Related Chronic Non-Communicable Diseases

Most Arab countries have faced marked changes in their demographic, socio-economic and health status during the last fifty years. These changes have been reflected in changes to the lifestyle of the population including access to modern amenities e.g., motor vehicles, electricity, refrigeration, television. The lifestyle changes have affected levels of physical activity and have also included introduction of a range of processed foods, resulting from increased commerce and interchange trade with Western and Asian countries. New foods and food ingredients have been introduced to the diet in the Arab countries to varying extents. Similar to populations in many developing countries, the populations of Arab countries are experiencing a nutrition transition characterized by replacement of traditional diets with diets higher in fat and refined and processed foods. These changes in dietary patterns and lifestyles have been associated with diet-related chronic diseases such as obesity, cardiovascular diseases, type 2 diabetes mellitus, cancer and osteoporosis [44–47].

#### 3.2.1. Overweight and Obesity

Obesity has become a major public health epidemic responsible for increased morbidity rate worldwide, including developing countries. Most evidence from several studies indicates that obesity is a major public health issue in Arab countries although it varies widely from one Arab country to the other [48]. Overweight and obesity have been reported as ranging from 25% to 38% among adult men, and 28% to 83% among adult women. Among school children (6–18 years) the prevalence of overweight and obesity was reported to be in the range of 18%–46.9%, among boys and ranged from 17.6% to 46.9% among girls (Table 3). Data on obesity in the Arab Region showed that even in middle and low income countries, the prevalence of obesity is high. The Arab Gulf countries showed the highest prevalence of obesity among adults, however countries like Egypt, Morocco and Tunisia have a similar trend in obesity. Although there was no national data on prevalence of obesity among Arab poor countries such as Djibouti, Somalia, Sudan and Yemen, indicators from cross-sectional studies in urban areas showed a trend to a high prevalence of obesity. For example, the prevalence of overweight and obesity among school children (6–12 years) in Khartoum city in Sudan was 14.8% and 10.5%, respectively [49].

Characteristically, obesity in most Arab countries was reported to be more prevalent among women, urban dwellers, married, non-smokers, the inactive and high socio-economic classes. The prevalence of overweight and obesity among women may be attributed in part to the high parity among women in most Arab countries [50]. In Saudi Arabia, for example, it was reported that the mean BMI increased significantly with parity in Saudi women; the mean BMI was 25.1 in women with no parity and this increased to 27.1, 29.8 and 31.7 in women with 1–2, 3–4 and more than 4 parities, respectively [51].

Unlike western countries, obesity in Arab countries is more prevalent among women in urban areas and in the higher social class. In Jordan, for example the prevalence of obesity was 56% in urban area compared to 44% in rural areas. Similar trends were found in Egypt, Morocco, Oman and Tunisia [52]. Lebanon was an exception as obesity was more prevalent among rural than urban women. Interestingly, obesity occurred more among unemployed than employed women in several Arab countries. In Kuwait, for example, the percentage of obesity in unemployed women was 47% compared to 34% in employed women. In Saudi Arabia, the proportions were 79% and 53%, and that in Tunisia the percentages were 24% and 15%, respectively [51]. This may be due to the fact that employed women are generally young. It is also believed that the exposure of employed women to the community at work may possible put pressure on them to take care of their weight [52].

Long periods watching television or using the internet, beliefs and attitudes toward obesity, physical inactivity, food advertisements, high intake of fast foods and an increase of food intake outside the home are also reported to be associated with obesity among children and adolescents in some countries of the Region [52]. There are no specific programmes to prevent and control obesity in Arab countries. Some activities to promote physical activity and reduce high energy intake do exist in some countries, but with limited effect [15].

Under-nutrition, especially stunting, may be another contributing factors for the high prevalence of overweight in the Region. In a study among five Arab countries, namely Djibouti, Libya, Morocco, Syria and Yemen, it was found that the risk ratio (RR) for overweight in stunted children ranged from 2.14 in Djibouti to 3.85 in Libya. Recent indicators showed that metabolic alterations in children suffering from under-nutrition in all tissues and body systems. These alterations are towards energy conservation and maintaining a low metabolism. Stunted children have impaired regulation of food intake and have higher susceptibility to the effects of high fat diets. When energy intake improves, deposition of fat over time may result [53]. The association between catch-up growth of infant and obesity in later life should also be considered. It was found that children who showed catch-up growth between zero to two years were fatter and have more central fat distribution at five years than other children. Mechanism that signal and regulate early catch-up growth in the postnatal period may influence associations between small size at birth and risk for disease in adulthood [54].

#### 3.2.2. Cardiovascular Disease (CVD)

Reliable and complete mortality data is not available for many Arab countries. In general data collected by the WHO on the East Mediterranean Region (which include all the Arab countries) shows that CVD and stroke are rapidly growing problems, and are major causes of illness and deaths in the Region, accounting for 25–31% of the deaths [1]. A review of 31 articles on stroke in the Arab countries revealed that the annual stroke incidence ranged from 27.5 to 63 per 100,000 population and the prevalence was between 42 and 68 per 100,000 population. Ischemic stroke was the commonest subtype in all series. Hypertension, diabetes mellitus, hyperlipidaemia, and cardiac disease were the commonest risk factors [70]. Estimate of mortality of cardiovascular and diabetes in Arab countries according to per capita income are presented in Table 4. Interestingly, the estimated mortality of these diseases was high in low per capita income countries than middle and some high per capita income countries. For example, the estimated of mortality of cardiovascular and diabetes diseases in Somali was 570.7 and 573.5 per 100,000 people among males and females respectively, compared to 267.8 and 245.5 per 100,000 people in Tunisia for males and females, respectively (Table 4). This indicates that non-communicable diseases are no more problems of rich countries, and poor countries may be more susceptible to these diseases.

Approximately 75% of CVD can be attributed to known risk factors. With changing lifestyles in the Arab countries, there is likely to be greater exposure to risk factors such as high blood pressure, diabetes, physical inactivity, smoking, obesity and diets high in saturated fat, leading to elevated serum cholesterol levels [47]. Increase in proportion of old people is another contributing factor for the high prevalence of CVD. Policies, recommendations and programs designed to control CVD in Arab countries should emphasize what is valuable in traditional diets, existing food supplies, and physical activity patterns [72].

#### 3.2.3. Type 2 Diabetes

Diabetes mellitus is a group of diseases characterized by high blood concentrations resulting from defects in insulin secretion, insulin action or both. Type 2 diabetes, (also known as non-insulin dependent diabetes mellitus, NIDDM), may account for 90% to 95% of all diagnosed cases of diabetes and is a progressive disease that, in many cases, is present long before it is diagnosed. Risk factors for type 2 diabetes in Arab countries include genetic and environmental factors, family history of diabetes, old age, obesity (particularly intra-abdominal obesity), physical inactivity, a prior history of gestational diabetes, and ethnicity [1,47].

Diet is an important determinant factor of obesity and also influences insulin resistance, so has an important role in the development of type 2 diabetes. With urbanization and concomitant changes to lifestyles and dietary habits as experienced in Arab countries, the chronic consumption of high energy, high fat diets, as well as low levels of physical activity, lead to changes in energy balance with conservation of energy stored as fat. Such excess energy intake per se promotes insulin resistance even before significant weight gain occurs. A diet high in energy and low in fiber promotes weight gain and insulin resistance even in low risk populations. Diet and physical activity are corner stone’s in treatment targeting control of type 2 diabetes symptoms and in preventing progress and medical complications of the disease [73].

Many studies on type 2 diabetes in Arab countries have been published in the last decade. The majority of these studies address clinical issues, with only a few epidemiological studies employing WHO methodology and diagnostic criteria for type 2 diabetes. In those Arab countries where such surveys have been conducted, the prevalence of diabetes in population samples 20 years and older, has been reported to be around 10–12% [74–76]. Results of studies on diabetes in populations of some Arab countries indicate that diabetes is considerably more prevalent than in the general population of Europe and North America. Another important finding reported in a majority of these studies is the low detection rate of type 2 diabetes. The percentage of under-diagnosed diabetes was estimated to be in the range of 40% to over 60% [1,72].

#### 3.2.4. Osteoporosis

Osteoporosis is a bone disease characterized by a low bone mass and deterioration of bone tissue leaving bones fragile and susceptible to fracture. Contrary to popular belief, it is not a normal part of aging and is increasingly recognized as one of the major public health problems facing postmenopausal women and aging individuals. Numerous studies have clearly revealed the association between a decreased risk of osteoporosis and adequate intakes of calcium and vitamin D, increased physical activity and cessation of smoking. These lifestyle factors have been reported to account for up to 30% of the variance in peak bone mass [77]. Many women of differing ages and elderly men are likely unaware of the risk factors which can be effectively mitigated by early preventative measures. In a study on knowledge of osteoporosis among Arab females it was found that there were many misconception regarding causes and risk factor for occurrence of osteoporosis. The highly educated women had relatively better knowledge on causes and factors related to osteoporosis. All women were dependent on television, relatives and medical doctors as main sources of information on osteoporosis. Books and radio were never considered as source of information [78]. Prevention of osteoporosis should start by making high risk groups and the general population aware of risk factors.

Osteoporosis will soon be a problem of greater importance in developing countries with an expected increase in life expectancy. In the Arab countries, the burden of this disease is also expected to increase with the steady growth of the ageing population [30]. Studies have evaluated bone mass density (BMD) in a number of Arab countries. Reference ranges were suggested for Lebanon [31,79,80]. Saudi Arabia [81,82] and Kuwait [83]. All these studies, conducted mainly on female populations, found a lower BMD among the population of these countries compared to the standard established for Caucasian populations, except in Kuwait, where the BMD reference range was similar to US/European reference data. Many of these studies revealed that risk factors for osteoporosis, female sex, age, menopause, and smoking, were similar to those known to influence BMD in other populations [84]. Further risk factors were identified as being characteristic to Arab populations such as high parity, prolonged lactation and vitamin D deficiency. These risk factors, prevalent in the region, may account for the reported lower BMD compared to European and North American women.

High prevalence of hypovitaminosis D in Arab countries is significantly more prevalent in women than in men. In women and in men, inadequate vitamin D intake and urban dwelling are independent predictors of hypovitaminosis D. Islamic clothing and high parity are additional predictors of hypovitaminosis D among women [30,34].

The above studies clearly show that osteoporosis is a growing public health risk in the Arab countries. Yet most of these countries have not yet initiated serious efforts to commence prevention programs on osteoporosis. Promoting physical activity among various age groups, encouraging intake of foods rich in vitamin D and calcium, and exposed to sunlight are among the most important measures to promote the health of bone.

#### 3.2.5. Cancer

Cancer registry data in the Arab countries are scanty, especially for those countries with large populations. However, available statistics indicate that cancer is a major health problem, representing 8–12% of total annual death in the Region. In males, the predominant cancer vary, with lung, urinary bladder and liver in first place, while for females throughout the region breast cancer is the main type. In both sexes, non-Hodgkin lymphomas and leukemia are relatively frequent, along with thyroid cancer in certain female population [85]. Data on estimated mortality of cancer in Arab countries indicated that high per capita income have less mortality rate due to cancer than middle and low income countries (Table 4). This may suggest that the etiology of cancer is not same among these countries. Environmental problems and contaminated food with carcinogenic ingredients may be more occurred in middle and low income countries than high income countries. The Arab Gulf countries (with high per capita income) have developed more advanced inspection systems for domestic and imported foods, the matter that reduced exposure to carcinogenic contaminated food [86].

It is well-documented that most of the cancers prevalent in the Arab countries can be linked to diet and change in lifestyle. There is also a belief that the changes in dietary habits in Arab countries, with low intakes of fruit and vegetables and high intake of fats, might be contributing to the high prevalence of some types of cancer. Obesity, inactivity, smoking and genetic factors are also important factors to the prevalence of cancer in this Region [72]. Given the recent increase in hubble bubble (*Arkila, Narkila, Shisha*) smoking by youth across the region, more stress needs to be placed on antismoking efforts [85]. Our understanding and knowledge of cancer as a public health problem in Arab countries is an area that needs to be improved over the coming decade.

### 3.3. Factors Contributing to the Prevalence of Nutrition-Related Diseases in Arab Countries

#### 3.3.1. Economic Factors

The Arab Countries will continue to highly rely on imported food, due to continue increase in population numbers and the limitations of agriculture and natural resources. According to the estimate of food dependence indicators, the Arab countries can be grouped into three categories: (1) Independent countries, including Jordan, Tunisia, Sudan, Syria, Iraq, Palestine, Egypt, Morocco and Yemen. (2) Intermediate between independent and dependent countries, including Algeria, Saudi Arabia, Oman and Libya. (3) Dependent countries such as Bahrain, Kuwait, Qatar and United Arab Emirates [87].

However, based on *per capita* income, Arab countries can be divided into three categories: (a) high *per capita* income countries such as Bahrain, Kuwait, Oman, Qatar, Saudi Arabia and United Arab Emirates; (b) intermediate *per capita* income countries such as Egypt, Jordan, Iraq, Lebanon, Libya, Tunisia, Morocco and Algeria; and (c) low *per capita* income countries such as Djibouti, Mauritania, Somalia, Sudan and Yemen. In general, the food situation in Arab countries has changed markedly during the last four decades (1965–2005). However, the change in food habits is not the same in the three groups of countries. The share of some food groups in total *per capita* energy intake consumption in the three income categories is presented in Table 5. In the high-income countries, the traditional diet, which consisted of dates, milk, both vegetables and fruits, whole wheat bread and fish, has changed to a more diversified diet, with an excess intake of energy-dense foods rich in fat and free sugars and deficient in complex carbohydrates, with the daily energy intake exceeding 3,000 kcal/*per capita*. Although sugar consumption is already high, it continues to rise and its contribution to the total energy intake ranges from 10% to 15%. The same trend is applicable to fat consumption (both vegetables and animal) that now comprises over 30% of the total energy intake [10,88].

The average *per capita* energy supply in the intermediate-income countries ranged from 2,800 to more than 3,000 kcal. Cereals contribute more than half of this calorie intake. Sugar consumption has also risen considerably and now makes up 8 to 15% of the total energy intake. Similarly, fat consumption has increased in several of these countries and comprises 20–30% of the daily energy supply. The change in habitual dietary intakes in these countries is mainly due to the shift to middle and upper social classes in the last three decades (1970–2000) following the economic boom and the development of industry and services. Most people now live in large cities whose are gradually adopting the eating habits and lifestyles of the wealthier classes elsewhere [10].

The average *per capita* energy supply in the intermediate-income countries ranged from 2,800 to more than 3,000 kcal. Cereals contribute more than half of this calorie intake. Sugar consumption has also risen considerably and makes up 8 to 15% of the total energy intake. Similarly, fat consumption has increased in several of these countries and comprises 20–30% of the daily energy supply. The change in habitual dietary intakes in these countries is mainly due to the shift to middle and upper social classes in the last three decades (1970–2000) following the economic boom and the development of industry and services. Most people now live in large cities whose are gradually adopting the eating habits and lifestyles of the wealthier classes elsewhere [10].

Low-income countries in the Arab region have the same food consumption characteristics as many poor countries in the world. The daily caloric intake is 2,000–2,800 kcal and cereals comprise more than 50% of total intake. It is worth mentioning, that in the large cities of these low-income countries, the higher social classes have similar dietary intakes to their counterparts in the intermediate-and high-income countries [10].

Rural poverty is one of the main problems linked to food security and diet-related diseases in the Arab countries. About 25% of Arab population is poor (based on national poverty lines), and 76% of these poor people are living in rural areas. The poor in the region are severely affected by price shocks, spending 35–65% of their income on food [89].

Water and land constraints are challenging for food security in Arab countries. About 75% of exploitable renewable water resources are taken out of the natural system and put to use, compared to 1–30% in other regions [90]. Additionally, there is a little or no potential for sustainable increase in water use in most Arab countries. As for Arab land, the expansion is slower when compared to other countries in the World [91].

#### 3.3.2. Food Prices Crisis

Arab countries are very susceptible to food price fluctuations because they are heavily dependent on imported food. Arab countries are the largest importers of cereals in the World, and they import at least 50% of food calories consumed [91]. Factors causing increases in food demand are growing faster in Arab countries than the rest of world. The population growth rate is estimated to be 1.7%, compared to 1.1% globally. The current income growth rate of Arab countries exceeds the global average (3.4% and 3% respectively). This means increased purchasing power of the people in Arab countries [92]. Urbanization is another important factor, compounding the effects of food prices, with urban population growth of 3% during 1990–2006, exceeding the global average of 2.2% [91].

The food-price shock during 2007–2008 had a significant impact on inflation in the Arab countries. This food shock was associated with an additional four million undernutritioned people in the Arab countries [90]. In 2007 the annual percent in food inflation in Yemen, Syria, Egypt, Djibouti, Jordan, Lebanon, Algeria and Morocco outpaced the changes in overall inflation. In addition to inflation, food price shocks directly affect trade and fiscal balances. Several Arab countries such as Egypt, Jordan, Syria, and Yemen have increased salaries for the public sector employees, and have increased food subsidies to support the poor. These measures forced oil-poor countries to reduce their expenditures on other essentials or increase borrowing, which have a negative long-term effect on their economies. Non-oil-exporting countries that are highly dependent on cereal imports such as Jordan, Lebanon, Morocco and Djibouti have fiscal and trade deficits that contribute to their economic hardship. As for oil-rich countries (mainly Bahrain, Kuwait, Oman, Qatar, Saudi Arabia and United Arab Emirates) have been able to absorb higher food prices due to their higher oil revenues. These countries have raised the public sector wages and implemented large food subsidy programmes without significant negative effect or fiscal strain. However, the decline of oil price and the continued increase in food price could affect fiscal and trade surpluses which may lead to chronic deficits in these countries too [89]. Poor families that respond to price shock by reducing calorics intake or by shifting consumption from healthy foods to cheaper, but less nutritious foods, which increase their exposure to undernutrition [10]. Shocks can also lead the poor to reduce investment in human capital. For example, poor families may discontinue preventive health care and withdraw children from school to generate additional income. [93].

#### 3.3.3. Change in Dietary Habits

Despite methodological difficulties involved in dietary studies of the various populations in the region, there is extensive documentation of the adverse effects of certain dietary patterns on the prevalence of nutritional disorders. Two types of dietary habits can be identified in most Arab countries. The first associated with the prevalence of diet-related non-communicable disease such as cardiovascular disease, hypertension, diabetes, obesity and cancer. The second type associated with the prevalence of under-nutrition especially growth retardation among children and micronutrient deficiencies.

##### 3.3.3.1. Dietary Habits Associated with Under-Nutrition

Studies on dietary habits linked with prevalence of under-nutrition in the Region are scarce. Some of these dietary habits are briefly discussed here. Weaning habits are considered to be one of the main factors affecting the health of infants in Arab countries. Many mothers introduce local foods, herbs and liquids as early as the first or second month of infant life. Bottle feeding is also early introduced at an early stage and this may lead to early cessation of breastfeeding and concomitant exposure of the infant to infections and diarrhea [2,3].

Inadequate intake of micronutrients is another important factor responsible for undernutrition, especially among children and adolescents. Indicators show that the daily intake of iron, calcium and vitamins D and C in the region are below the recommended dietary allowance (RDA). Iron deficiency anemia, for example, is highly associated with low intake of iron-rich foods. This is exacerbated by a low intake of foods that enhance iron absorption such as those rich in vitamin C and a high consumption of compounds that inhibit iron absorption include phytates (found in legumes and whole wheat flour), polyphenols (found in tea, coffee, cocoa and some spices and vegetables) and calcium. Vitamin C is mainly found in fruit and vegetable and there is good evidence that the consumption of fruit and vegetable is low in all age groups in most Arab countries. Tea consumption in the region is relatively high, which may contribute to the inhibition of iron absorption in poor families who depend on a plant-based diet [88]. However, most of the recommendations suggested not to drink tea directly after meal, but to delay the intake after two hours of consuming the meals. Also tea intake in between meals has almost no effect on the absorption of iron [94].

Skipping breakfast or the intake of a poor nutritional value breakfast is common among children and adults in the region. It has been suggested that skipping breakfast decreases the possibility to consume the recommended daily allowances of micronutrients. In general, the percentage skipping breakfast is higher among females then males. Skipping breakfast by school children in some Arab countries was estimated to range from 28% to 70% depending on age, sex and social class [15].

##### 3.3.3.2. Dietary Habits Associated with Diet-Related Non-Communicable Diseases

###### 3.3.3.2.1. Fat and Cholesterol Intake

Excessive dietary fat intake has been linked to increased risk of obesity, diabetes, coronary heart disease (CHD) and certain types of cancer. The mechanisms by which these are linked are complex, varied and, in many instances, not clearly understood. Elevated levels of serum cholesterol and low-density lipoprotein (LDL) constitute major risk factors for atherosclerosis and CHD. The degree of risk of these and other factors may vary according to, *inter alia*, the type and level of fatty acid intake, percentage of energy from total fat, dietary cholesterol, lipoprotein levels, intakes of antioxidants and dietary fiber, activity level and health status. Low-fat diets are often lower in cholesterol and higher in antioxidants and dietary fiber than high fat diets [95].

During the period 1970–2005, there has been in increase in *per capita* energy and fat supplies in most Arab countries. The increase in calorie supplies during this period ranged from 10% in Sudan to 40% in Egypt. Data from food balance sheets prepared by FAO showed that a high percentage of these calories came from animal foods. This is particularly true in high-income countries. Daily *per capita* fat supplies showed impressive increases, compared to the total supply of calories. The percent of increase ranged from 13.6% to 50%. Although some low and middle income Arab countries showed a marked increase in *per capita* fat supplies, most of the fat comes from plant origin, exception are Sudan and Somalia, where the intake of animal food is very high, and consequently the intake of fat coming from animal origin. The increase in *per capita* fat supplies in the Arab Gulf countries is also largely of animal origin [10]. Limited studies focused on type of fat intake in the Arab countries. In Egypt, Mahmood [96] found that 37% of fat consumed by Egyptian women was saturated fat. In Bahrain, Gharib and Rasheed [97] found that 36% to 50% of school children exceeded the limit of saturated fat intake. The polyunsaturated:saturated fat ratio was at unacceptable level of 0.6 for males and females.

Information on cholesterol intake in the region is at most scanty. In a study among obese adults in Kuwait it was found that the mean daily intake of cholesterol ranged between 600 to 4,900 mg, with an average of 1,676 mg. Several studies have indicated that a high percentage of adults in the region have elevated blood cholesterol. In the United Arab Emirates, the elevated cholesterol ranged from 66 to 84% in adult men aged 35–49 years. In Lebanon, about 18% of men and 23% of women had blood cholesterol levels of more than 240 mg/dL [88].

According to WHO/FAO recommendations, the upper limit of dietary fat intake for active individuals, who are in energy balance, is up to 35% of their total energy intake come from dietary fat if their intake of essential fatty acid and other nutrients is adequate. The level of saturated fatty acids should not exceed 10% of the energy they consume. Sedentary individuals should not consume more than 30% of their energy from fat, particularly if it is high in saturated fatty acids which are derived primarily from animal sources [77].

###### 3.3.3.2.2. Fiber Intake

The relationship between dietary fiber intake and management of some chronic diseases including hyperlipidemia, diabetes and obesity has been under investigation for many years. With regard to hyperlipidemia, consumption of fiber has been shown to reduce blood cholesterol levels in several studies. Studies on the intake of fiber in the Arab countries are few. This is mainly due to the lack of information on the fiber content of several foods consumed, as well as to general neglect of the role of fiber in health and disease in nutritional surveys. However, since fiber is found only in the carbohydrate portion of the diet, it is widely accepted that the level of fiber in the Arab diet is decreasing due to evidence of a decrease in the percentage of dietary intake from carbohydrate. Additionally, foods in the region are becoming increasingly processed with the result that grain products tend to be more refined and thus lose their fiber content. A further decrease in fiber intake takes place with a decrease in the consumption of whole grains. For example, sorghum and millet which are usually unrefined (and therefore keep much of their fiber) are becoming less important in the diets of poor Arabic countries, and are being replaced by refined wheat flour [88].

In Saudi Arabia, it was found that the average daily consumption of fiber was almost equal among both sexes (13.2 and 13.4g for males and females, respectively). The national nutrition survey in Saudi Arabia reported a higher figure with a daily average intake of fiber of 24.4 g. The main contribution to fiber intake came from vegetables and their products (31%), followed by cereal and their products (26%), and fruits and their products (25%). However, the food preparation methods, such as peeling vegetables and fruits, and using wheat flours with low extraction rates in breads, are contributing to a lower intake of fiber than those reported in these studies [88].

Fresh fruits and vegetables are considered rich sources of dietary fiber in addition to mineral, vitamins and antioxidants. There is good evidence that intake of fruit and vegetables participate in the prevention of certain chronic diseases such as obesity, hypertension, CVD and some types of cancer. The trend in consumption of these foods can be a good indicator for the occurrence of these diseases. Based on WHO Stepwise surveys in six Arab countries (Egypt, Jordan, Iraq, Kuwait, Saudi Arabia and Syria), it was found that the low intake of fresh fruit and vegetables (below five servings/day) ranged from 79% in Egypt to 95.7% in Syria [1]. Increasing individual fruit and vegetable consumption up to 600 g per day (the baseline of choice) could reduce many non-communicable diseases, especially cardiovascular disease and some types of cancer. This is creates the need of more emphasis on dietary risk factors in public health policy in order to tackle the increase in non-communicable diseases in the Region [98].

The intake of fiber-rich foods by children and adolescents in most Arab countries is alarmingly low. The dietary habits of school children and adolescents in the region are characterized by low intake of fresh fruits, vegetables and milk and a high intake of carbonated beverages and fast foods. In general, the food habits of Arab adolescents, particularly in urban areas, have become similar to those reported for Western communities in relation to snacking patterns and consumption of fast foods. These changes in food habits may in part explain the increase in diet-related chronic diseases in some Arab countries [15].

###### 3.3.3.2.3. Salt Intake

The role of salt or sodium intake, principally as sodium chloride, as a cause of hypertension has been under study for many decades. Although study results were not consistent, there is a general agreement that salt intake should be reduced for patients with hypertension, diabetes and heart disease. The estimated daily requirements for salt are no more than 8–10 mmol of sodium or 500 mg of sodium chloride per day. Indicators showed that the intake of sodium in this region exceed these requirements. In Jordan, for example, the intake of sodium was 10 times higher than the minimum requirement. However, the minimum recommended intake of sodium for the people in the Arab countries is probably higher than for Western countries recommendation due to the hot and humid climates in these countries. Data from food composition tables for the Arab countries showed that sodium content in the diet of the Arab countries is high. This is due to several reasons: high use of table salt, spices and pickles, and the salinity of water (in some countries). Additionally, the low intake of fruit and vegetables, may have an impact of low intake of potassium, which has a protective effect against hypertension. This means that the risk factor for hypertension in Arab Region is not only affected by high salty food intake, but also by low potassium food intake. Therefore, nutritional education should focus on healthy preparation of food at home, including lower use of salt and fat [88].

#### 3.3.4. Ramadan Fasting

Ramadan is the ninth month of the Islamic calendar (Hijra), when Muslims abstain from food and drink from dawn until sunset. The common practice is to eat two meals, one before dawn and one after sunset, rather than the three meals normally consumed during other months [99]. In general, food consumption patterns are changed dramatically, as the intake of meat and traditional sweets is increased. It is highly believed that these changes may negatively impact the health of Muslims during this month. The effect of Ramadan fasting on body weight and composition remains a controversial subject. Some studies have demonstrated a reduction in body weight and other studies showed a gain in body weight [100]. In Saudi Arabia, Al-Numair [101] found that there was a significant decrease (p < 0.05) in the level of serum glucose, serum total protein and serum triglyceride, and in contrast, there was a significant increase (P < 0.05) in serum uric acid. There was no significant changes in the levels of serum total cholesterol and LDL. Hallak *et al.* [99] studied the changes in body weight and blood lipids during Ramadan fasting in Syrian men on hypocaloric diets. By end of Ramadan, they found that body weight, blood triglycerides and HDL-C had decreased significantly (P < 0.05), while LDL-C had increased, and total cholesterol had not changed compared with baseline values. During the daylight hours of Ramadan fasting, especially during summer, Muslims may dehydrated, but is not clear whether they are chronically hypohydrated. Available studies showed no detrimental effects on health attributed to water unbalance during Ramadan [102].

#### 3.3.5. Environmental and Social Factors

Undernutrition may be associated with poverty, high prevalence of infectious diseases, lack of access to clean water, poor sanitation and limited access to primary health care. These factors are more common in low-income countries such as Djibouti, Mauritania, Somalia, Sudan and Yemen. Food availability for many households in these countries is constrained and many depend on the market for their grain access. Purchasing power is very low, therefore, many families reduce their food intake. Some families skip one of the main meals to save food [3].

Diarrhoea, malaria, acute respiratory infection and dysentery are highly prevalent in poor Arab countries. Illness due to these diseases interferes with utilization of nutrients, even if the consumption of food is in sufficient quantity. The impact of infection, especially diarrhoea, on the growth of children in developing countries has been well researched. Mechanisms by which diarrhoea and other infections causes malnutrition include a decreased intake of food due to anorexia or withholding of food, decreased nutrient absorption, increased metabolic requirements, and direct nutrient loss [12]. In Sudan diarrhea and respiratory infections were significantly associated with increase in children’s growth patterns, even after adjusting for age, gender, socioeconomic status, dietary habits and previous morbidity. Attained height was on average 17 mm lower (95% CI, 15–19) for children with diarrhea than healthy children. The risk of being stunted 6 months later was 1.38 times (95% CI, 1.70–1.59) greater among normally-nourished children with diarrhoea than among those without it [103].

Access to clean water supply in many poor Arab countries varies from village to village, but in general availability of safe water for drinking and food preparation is a concern. In addition, sanitation and hygienic practices are low. Many diseases, including acute diarrhoea in children, typhoid and other bacterial illnesses are caused by contaminated water and food. The high prevalence of diarrheal diseases is attributes to unsafe water supply, inadequate sanitation and hygiene. Safe water, basic sanitation, and waste management combine to form the safe environment needed for delivery health care, and consequently improve nutrition status of the people [104]. The ongoing conflicts in Sudan and Somalia lead to the displacement of large populations into temporary camp settlements with overcrowding and rudimentary shelters, inadequate safe water and sanitation and increased exposure to disease vectors [105].

Food contamination is of particular concern for infants and young children who are at critical phases of growth and development [2]. A study to detect the presence of aflatoxin B1, in 60 samples of peanut butter available in the market of Khartoum state, Sudan, found that aflatoxin B1 was detected in all samples. Traditionally prepared samples showed the highest proportion of aflatoxin B1, which was above the internationally regulated tolerance levels (5–20 ppb). The range of aflatoxin B1 in the samples studied was 17–170 ppb. The results indicated that peanut butters prepared by the street sellers and distributed by retail stores in Khartoum were hazardous to human health [106].

Access to and utilization of the current primary health care (PHC) system may buffer the child against the risk of undernutrition and may decrease the risk of complications and resultant morbidity. The current primary health care system in low income and some of the middle income Arab countries mainly covers urban areas and a few rural communities. Despite the continuous training of health assistants, nurses, and other health workers, the gap between the ideal situation and services in the field is still wide. Many areas have no nutritionists or health staff trained in nutrition. In Sudan, for example, the primary health care units, in most cases, are 2–7 km away from the villages. Women use a donkey or go on foot to visit these units. Therefore, women only go to PHC when the child or a family member has a severe illness [107].

Parasitic infestation may also contribute negatively to the nutritional status of the people in some Arab countries. Studies have indicated that parasites were highly prevalent among children [108,109] and adults [110] in the Arab Region. In Palestine, it was found that 32% of patients attending hospitals had a parasitic infestation. The most common parasites were: *Entamoeba histolylica* (54%), *Cardia lamblia* (23%) and *Ascaris lumbricoides* (20%) [111]. The prevalence of parasites in some vegetables commonly consumed in South Westren of Saudi Arabia ranged from 13% to 28%. *Ankylostoma duodenale*, *Entameba coli*, *Ascaris lumbricoides* and *Blastocystis hominis* were the most common isolated parasites [112]. The high prevalence of intestinal parasitic infections in the oil-rich Arab countries could be attributed to importation of these diseases by immigrant labour brought in as domestic help who are coming from endemic countries such as India, Srilanka, Indonesia and Philippines [113].

Many diseases prevalent in Arab countries such as cardiovascular mortality and respiratory illness could be linked to climate fluctuations (in addition to other known risk factors) due to heat waves, as well as altered transmission of infectious diseases and malnutrition due to crop failure. The total estimated deaths for CVD, diarrhea, malaria, floods and malnutrition attributes to climate change in 2000 compared to baseline climate of 1961–1990 in Arab countries ranged from 5–10 per million [114]. However, studies on the role of climate change in disease mortality in the Arab region are at most scanty. The role of climate change in diseases morbidity and mortality should be a subject for further research in Arab countries.

#### 3.3.6. Inactivity

Changes in lifestyle and socio-economic status in this region have had a significant effect on physical activity. In most countries of the region, with the availability of cars, electrical home appliances and more involvement in office work, life has become more sedentary, and the pattern of physical activity during the last two decades has decreased steeply. In Egypt, it was found that regular physical activity was the least performed leisure activity during a typical day. Only 2% of adults (20–70 years) were reported as practicing regular physical activity during a typical day, 8.5% during the weekend and 2.5% during their annual leave [115]. A study among Saudi adults using the International Physical Activity Questionnaire (IPAQ) showed that over 43% of Saudis did not participate in any type of moderate-intensity physical activity lasting for at least 10 minutes. More than 72% of subjects did not engage in any type of vigorous-intensity physical activity lasting for at least 10 minutes. Females were more engaged in moderate physical activity than males, whereas male participated more in vigorous activity than females. Based on the three activity categories established by IPAQ, 40.6% of Saudi’s were inactive, 34.3% were minimally active and 25.1% were physically active [116].

Physical activity was lower among those who were married, worked in private sectors, worked two shifts, were less educated or who had only one day off during the week. Time constraints seemed to be the major contributing factor to inactivity, while maintaining health and losing weight were the most important reason for being physically active among Saudi men [116]. Using a WHO STEPwise survey in seven Arab countries (Egypt, Iraq, Jordan, Kuwait, Saudi Arabia, Sudan and Syria) it was found that low physical activity among adults ranged from 32.9% in Syria to 86% in Sudan [1].

Most studies in the Arab countries focused on barriers to practicing physical activity in women. This may be due to difficulties faced by females in practicing exercise in some Arab culture, as males in general have more freedom and places to practice sport and other recreational activities. A review of 10 studies investigating barriers to practicing physical activity and sport in six Arab countries (Egypt, Jordan, Bahrain, Qatar, Sudan and Iraq), indicated that the barriers can be divided into five categories: economic, social, psychological, cultural and environmental. Women’s commitments to work/home, physiological conditions (pregnancy and lactation) and lack of places and facilities for women to practice exercise were the main barriers perceived by the women studied [51].

#### 3.3.7. Political and Humanitarian Crisis

The conflicts and political situation in some Arab countries have a great negative impact on the nutritional status of people, especially women and children. This is particularly true in Sudan, Palestine, Iraq and Somali. According to the report of the Personal Humanitarian Envoy of the United Nations Secretary-General a serious and mounting humanitarian crisis is occurring in the West Bank and Gaza. The crisis is provisionally evidenced by rising level of malnutrition among children, high levels of poverty and unemployment, deteriorating health conditions and an increasing exhaustion of the coping mechanisms [117].

The siege in Gaza has led to a steady rise in chronic malnutrition among the 1.5 million people living in the strip, according to a leaked report from the Red Cross [118]. The crippling economic blockade of the Gaza Strip has impacted the various aspects of public life in this coastal region. The Hamas-run Palestinian Health Ministry reported that 70% of Gaza’s 1.5 million residents suffer from anemia, including 44% of pregnant women. Malnutrition among Palestinian children has also increased over the past 11 months, affecting more than 10% of Gaza’s children under the age of 18 months. The inability of the majority of Palestinian households to purchase basic food items has increased the magnitude of this health problem. A large number of household in Gaza are currently unable to afford essential food items as the siege on the Gaza Strip continues with additional restrictions on food and fuel. A recent malnutrition survey, conducted by the Ard Al-Insan health organization in Gaza City, revealed that around 10.4% of household in Gaza City and in the northern and southern Gaza Strip suffer from chronic malnutrition with many of them facing growth difficulties in terms of weight and length [119].

Apart from dodging bombs and bullets in their schools and neighborhoods, children in Iraq are suffering from worryingly high levels of malnutrition. Poverty and insecurity are the main causes of deteriorating diet of Iraqi children. However, violence and displacement of hundreds of thousands of people making it very difficult for monthly food rations to reach the families that need them most. Many Iraqi children are suffering from hidden hunger (deficiencies in essential vitamins and minerals), making the children more vulnerable to illness and less likely to thrive at school. The rate of exclusive breast feeding was decreased dramatically among Iraqi mothers, and infant formula is widespread. This increase the risk of illness, especially diarrheas which contribute to under-nutrition. The problem has become more complicated with absence of safe water and basic sanitation [120].

The deteriorating security and increasing violence inside Iraq has promoted an unprecedented exodus of Iraqis. More than 2 million have fled to the neighboring countries. Syria is currently hosting about 1.5 million Iraqis. Rapid assessments carried out on these migrant Iraqis showed that lack of awareness of services provided by United Nations Organizations is overarching issue which affects access to education, nutrition, and health and child protection services. A second sweeping issue is the rapid depletion of resources that Iraqis are facing due to unemployment. Displaced Iraqis can only work informally in Syria. Most, therefore, are livings off saving, uncertain remittances from relatives in Iraq, or money gained from selling their assets [121].

### 3.4. Programmes to Prevent and Control Nutrition-Related Diseases

Although many Arab countries have established a Nutrition Plan of Action to overcome nutritional problems, as recommended by WHO/FAO [122], none of these countries have completely implemented their plans. Only a few of the activities mentioned in the Plans were carried out. Several factors contribute to the poor implementation of nutrition programmes, such as the lack or absence of nutritional surveillance data, inadequate training for medical professionals and paramedical on dietary management of nutritional problems, a focus on curative procedures for nutritional problems rather than preventive measures, inadequate health information systems, lack of studies related to ecological factors associated with nutritional problems and lack of work on assessing the cost-effectiveness of various nutrition intervention.

In general the most common nutrition programmes carried out in this region are: food fortification, nutrition education for the public, school feeding, breastfeeding support and food subsidies. Food fortification is focused on flour fortification (mainly wheat flour) with iron and folic acids, to prevent and control iron deficiency anaemia. Some countries fortify salt with iodine as a measure to prevent iodine deficiency disorders (IDD). However, studies to evaluate the effectiveness of food fortification in the prevention of nutritional deficiencies are few, and existing studies have several methodological deficiencies [123].

The need for food-based dietary guidelines (FBDG) has been emphasised by FAO/WHO [124]. In order to prevent and control of diet-related diseases the Arab Center for Nutrition has established food-based dietary guidelines for the Arab Gulf countries, with special emphasis on protection against chronic diseases [125] (Box 1). However, these guidelines can be used in all Arab countries as it covers the main advices to prevent and control diet–related diseases in the Region. These guidelines were developed through 6 steps: determine the purpose and goals for establishing FBDG, characteristics of FBDG, determine the food consumption patterns, review the food and nutrition status, determine the lifestyle patterns that are associated with diet–related diseases and formulating the FBDG [124,125].

Box 1 **Food-based dietary guidelines for the Arab Gulf countries [125]**

(To prevent and control diet-related diseases)

1. Eat a variety of foods everyday.
2. East an adequate amount of fruit and vegetables daily.
3. Eat meat, fish, chicken, legumes and nuts regularly.
4. Make sure that our daily diet contains an adequate amount of cereals and their products.
5. Consume an adequate amount of milk and products everyday.
6. Reduce the intake of food rich in fat.
7. Reduce the intake of food and drink high in sugar.
8. Reduce the intake of sodium and salty foods.
9. Consume an adequate amount of water and other liquids daily.
10. Maintain an appropriate weight for your height.
11. Make physical activity a part of your daily routine.
12. Do not smoke and reduce the risk of exposure to smoking environment.
13. Avoid drinking alcoholic beverages.
14. Ensure safety of food eaten.

Control programmes for iodine deficiency disorders (IDD) are not usually targeted to specific age or sex groups, e.g., women or children, but rather to whole populations. Successful IDD control programmes would result in the promotion of iodine status in, along with other groups, women, adolescent girls and children. Consequently, improvements in their physical and mental health will occur. The Region has been very active in this area over the past two decades, with support from the World Health Organization, the United Nations Children’s Fund and the International Council for the Control of iodine Deficiency Disorders. Not all countries in the Region, however, have national control programmes. In Tunisia, IDD has been officially declared by the World Health Organization to be under control, and in Jordan, Lebanon, the Syrian Arab Republic and Yemen it is said to be almost under control. Seventeen of the remaining countries have ongoing programmes for universal salt iodization and 16 have appropriate legislation for this. In the Region as a whole, about 51% of households currently consume iodized salt [126].

Nutrition education programmes for the public are usually carried out through the mass media, especially television, booklets and newspapers. Although, studies on evaluation of the impact of nutrition education programmes on nutrition behavior are limited, available evidence has shown that these programmes often play a small role in preventing nutritional diseases. This is mainly due to the high rate of illiteracy in some countries, the absence or lack of specialized staff in nutrition education, incorrect selection of target groups and inadequate information provided through the programme [2].

School feeding programmes in Arab countries are highly depending on regulations for foods provided by school canteens. These regulations are focused on nutritive values of food and portion sizes. In general, sweets and carbonated beverages are forbidden to be provided in schools. Some countries forbid potato chips and foods high in salt and fat. It is worth mentioning that the implementations of these regulations vary from country to country and from school to school within the same country. The Arab Gulf countries, (Bahrain, Kuwait, Oman, Qatar, Saudi Arabia and United Arab Emirates) have established school feeding guidelines as one of preventative measures for obesity and to promote healthy eating among school children. Also, some Arab countries have no specific regulations for foods to be sold in school canteens and all kinds of foods are provided. Another important factor is that relatively high percentages of children bring their foods from home, and many of these foods are rich in energy, salt and fat [127].

Programmes to support breastfeeding are considered one of the most successful nutrition programmes in the Arab countries. The UNICEF and WHO programmes, such as baby-friendly hospitals, steps to successful breastfeeding and the Code of Human Milk Substitute have been widely implemented in the region. However, one of the main obstacles to supporting extended breastfeeding is the early introduction of foods and liquids to the infant (from the first month). This practice has been documented in all Arab countries, and is highly influenced by educational level, age and employment of mothers. Taboos and cultural beliefs and attitudes also have an important role in the introduction of food at an early stage of infant life in several countries of the region [126].

The Baby Friendly Hospital Initiative, launched jointly by the United Nations Children’s Fund and the World Health Organization in 1991–1992, with the aim of supporting and promoting breastfeeding in different countries, has been successful in its goals and objectives in the Region. The national breastfeeding authorities control the relevant measures and programmes using global criteria. As a result of these efforts, more mothers now breastfeed their infants in the Region as a whole. The proportion of children exclusively breastfed for 3 months is over 40%. The proportion breastfed for 6–9 months with complementary feeding increased from 38% in the period 1990–1996 to 45% in the period 1995–2002, and about one third are now breastfed 20–23 months [126].

Most Arab countries have food subsidy policies that keep the price of staple foods within the purchasing power of the majority of population. The main subsidized foods are rice, wheat, sugar, vegetable oils, fat and red meat. It is believed that this policy may encourage some people to overconsume these foods (which are high in dietary energy) and might be one underlying factor for the increasing obesity problem. On the other hand, subsidization of red meat and animal fat might contribute to an increase in the prevalence of coronary heart disease and some types of cancer, if its consumption is high and over a long period [128]. One suggestion that has been indicated for food subsidy policy is that the government should find the possibility to subsidize some healthy foods such as fruit, vegetables and fish. However, this suggestion needs further investigation as the price of these foods is unstable and highly influenced by seasonal variations.

In 2007, the third Arab Conference on Nutrition was held in Abu Dhabi, United Arab Emirates, and released the Abu Dhabi declaration to promote healthy nutrition in Arab countries [129]. The declaration included several important activities to prevent and control nutritional problems in the Region (Box 2).

Box 2 **Abu-Dhabi Declaration to promote healthy nutrition in the Arab countries [129]**

In order to promote healthy nutrition in the Arab countries the following activities should be taken into consideration:

1. Training of health workers and related fields in assessment, prevention, and control of nutritional problems with special emphasis on training of physicians, nurses, school teachers and social workers.
2. Reviewing and evaluating the current curricula in both government and private schools in order to update the information related to nutrition and linking this information with the local and Arab situation.
3. Encouraging university academic carriers to write textbooks in Arabic and translate related academic publications, through financial support from international and regional organizations, as well as private sectors with special emphases on multi-author publications.
4. Providing opportunity to young local nutritional specialists to participate in nutrition activities and programmes in order to prepare them to take the leadership in the future.
5. Updating and developing the current academic curricula, especially in colleges of agriculture and home economic in the region.
6. Encouraging establishment of a nutrition unit or section in the preventive health or public health departments in the ministry of health to promote preventive health programmes.
7. Integrating nutrition in a broad way in university curricula as well as in medical, health sciences and nursing schools.
8. Establishing legislations and regulations for commercial advertisements in mass media, especially for those related to nutrition, health and physical activity.
9. Working with both public and private sectors to develop and improve food products to provide nutritious foods.
10. Encouraging health and nutrition specialists to participate in workshops, training courses, conferences which are carried out in various Arab countries, to exchange knowledge and experiences. This can be done through providing short fellowships from public and private sectors.
11. Supporting awareness programmes to promote healthy nutrition and healthy lifestyle through various mass media.
12. Encouraging studies and research in food and nutrition through financial and technical supports, with more focusing on joint research among several countries in the Arab region.
13. Conducting regional conferences on food, health and nutrition on regular basis, especially the Arab Nutrition Conference and the Arab Conference on Obesity and Physical Activity, which are carried out every three years in one of the Arab countries.
14. Preparing or updating the national nutrition plan of action which should be a part of the national health plan in each country.
15. Encouraging and establishing nutrition societies in each Arab country to support and coordinate the nutrition activities.
16. Supporting the therapeutic nutrition activities in all hospitals throughout the Arab region by preparing uniform food guidelines, portion size and food composition tables to suit the Arab food habits and culture.
17. Developing and improving the food control activities to provide safe foods for the public.

## 4. Conclusions

Arab countries have experienced dramatic changes in health and nutritional status over the past five decades. Growth retardation among young children and iron deficiency anemia are the most common nutritional problems in all these countries. Diet-related chronic diseases such as obesity, diabetes, hypertension, cardiovascular disease and some types of cancer are emerging health problems in most countries of the region. There are several social, economic, lifestyles and nutritional factors contributing to the prevalence of these nutritional diseases [1,73]. Although many countries in the region have prepared a national plan of action for prevention and control of nutritional problems, none of them have put these plans of action into practice. However, there are several scattered activities are carried out in the Arab countries to prevent and control of nutritional problems. More attention should be given to the policy makers of the region to improve their awareness about the importance of the prevention and control of nutritional problems for the improvement of the overall health status of the community. The misguided concept that the health sector alone is responsible for overcoming nutritional problems is widespread in Arab countries. Coordination and cooperation in nutrition activities between the health and other sectors is therefore weak and does not exist in some countries. The causes of nutritional problems require a clear understanding of the interrelationship between health, agriculture, social, economic and political factors in order to develop multi-sectoral programmes. Planning for these programmes should, however, be done with care and developed within the local context, situation and resource capacities of the Arab countries.

## Figures and Tables

**Table 1:** Prevalence of under-nutrition in children under the age of five years (%) in the Arab countries.

Country	Latest Survey year	Underweight		Stunting		Wasting	
		Moderate	Severe	Moderate	Severe	Moderate	Severe
		Less than −2 s.d	Less than −3 s.d	Less than −2 s.d	Less than −3 s.d	Less than −2 s.d	Less than −3 s.d
Algeria	2006	3.7	0.6	11.3	3.0	2.9	−
Bahrain	1995	8.7	1.8	9.7	2.7	5.3	−
Djibouti	2006	28.9	10.3	32.6	19.7	20.7	−
Egypt	2005	6.2	1.0	17.6	6.4	3.9	0.9
Iraq	2006	7.6	1.4	21.4	7.5	4.8	
Jordan	2002	4.4	0.5	8.5	1.6	2.0	0.4
Kuwait	1996	9.8	2.9	23.8	11.8	10.6	2.7
Lebanon	2004	3.9	−	11.0	−	5.4	−
Libya	1995	4.7	0.6	15.1	4.5	2.8	0.4
Mauritania	2000–2001	31.8	9.8	34.5	16.5	12.8	3.3
Morocco	2003–2004	10.2	2.0	18.1	6.5	9.3	2.5
Palestine	2006	2.9	0.4	10.2	3.0	1.4	−
Oman	1998	17.8	1.3	10.4	1.6	7.2	0.4
Qatar	1995	5.5	−	8.1	−	1.5	−
Saudi Arabia	1996	14.3	2.8	19.9	6.8	10.7	2.2
Somalia	2006	35.6	11.6	37.8	20.5	11.0	
Sudan	2000	40.7	14.7	43.3	23.7	15.7	3.8
Syria	2006	9.7	1.8	22.4	10.1	8.6	−
Tunisia	2000	4.0	0.6	12.3	3.4	2.2	0.5
U.A. Emirates	1995	14.4	3.2	16.7	6.8	15.2	3.8
Yemen	2000	45.6	15.2	53.1	30.9	12.4	3.0

Source: UNICEF [3], FAO [10], Mason *et al.* [11].

**Table 2:** Prevalence of micronutrient deficiency (vitamin A deficiency, anemia and Iodine deficiency) in the Arab countries (2000).

Country	Exophthalmia (night blindness + Bitors spot) children 0–72 month%	Vitamin A deficiency (serum retinol < 0.7 mmol/L children 0.72 month) %	Anemia non-pregnant women 15–40 years %	Anemia (pregnant women) %	Anemia (children 0–59 month) %	Iodine deficiency %
**Algeria**	1.0	28.9	31.3	35.2	37.6	16.7
**Bahrain**	−	−	36.2*	41*	−	−
**Egypt**	0.9	27.1	28.0	46.1	30.5	11.9
**Iraq**	1.4	41.7	40.1	26.2	36.3	24.6
**Jordan**	0.3	19.3	29.3	35.7	27.2	10.8
**Kuwait**	0.1	15.8	12.3	35.1	4.7	−
**Lebanon**	0.4	19.9	24.1	22.3	20.5	11.0
**Libya**	0.5	19.3	23.5	−	20.3	10.1
**Mauritania**	1.7	17.4	42.0	40.0	73.8	−
**Morocco**	1.1	29.2	34.0	39.3	45.0	−
**Oman**	−	−	−	−	60**	−
**S. Arabia**	0.5	20.9	18.6	23.1	18.5	−
**Somalia**	2.0	25.1	53.7	40.0	78.4	12.6
**Sudan**	1.6	35.8	44.3	45.0	70.2	11.5
**Syria**	0.6	22.0	30.1	40.7	39.5	27.1
**Tunisia**	0.8	21.5	27.0	30.5	32.2	09.1
**UAE**	0.1	13.7	10.5	35.1	1.4	−

* 1995,

** 1992. Source, Mason *et al.* [11].

**Table 3:** National prevalence of overweight and obesity among children, adolescents and adults in selected Arab countries.

Country	Children and Adolescents						Adults (15+ years)				
	Date of Survey	Age (years)	Sex	Overweight (%)	Obesity (%)	Ref.	Date of Survey	Sex	Overweight (%)	Obesity (%)	Ref.
**Bahrain**	2006	15–18	**M**	15.8	13.7	55	2007	**M**	34.8	32.3	63
			**F**	17.4	19.4			**F**	35.1	40.3	
**Egypt**	2004	10–18	**M**	11.5	6.5	56	–	**–**	–	–	–
			**F**	15.2	7.7						
**Kuwait**	2006	10–14	**M**	29.3	14.9	57	2007	**M**	38.9	39.2	64
			**F**	32.1	14.2			**F**	28.9	53.0	
**Lebanon**	1995–1996	10–19	**M**	26.9	7.7	58	1995–1996	**M**	43.4	14.3	58
			**F**	14.7	2.9			**F**	30.6	15.5	
**Libya**	–	–	**–**	–	–	–	2000	**M**	19.2	5.8	65
								**F**	21.1	7.1	
**Morocco**	–	–	**–**	–	–	–	1995–1999	**M**	28.0	05.7	44
								**F**	33.0	18.3	
**Oman**	–	–	**–**	–	–	–	2000	**M**	30.6	15.5	66
								**F**	27.2	22.3	
**Qatar**	2003–2004	6–9	**M**	16.3	3.5	59	–	**–**	–	–	–
			**F**	15.5	2.8						
**Palestine**	–	–	**–**	–	–	–	2002	**F**	–	10.9	67
**S. Arabia**	2005	5–12	**M**	19.9	7.8	60	2005	**M**	43.0	31.5	68
			**F**	19.2	11.0			**F**	28.8	50.4	
**Tunisia**	2005	15–19	**M**	17.4	4.1	61	2005	**M**	51.7	37.0	69
			**F**	20.4	4.4			**F**	71.1	12.3	
**UAE**	2005	10–19	**M**	21.2	13.2	62	–	–	–	–	–
			**F**	21.3	11.0						

**Table 4:** Estimate of mortality of Cancers, Cardiovascular and Diabetes diseases (age-standardized death rate per 100,000) in Arab countries according to per capita income.

Country	Cancers		Cardiovascular Diseases and Diabetes		Latest Year of Date*

	Males	Females	Males	Females	
	**High Per capita income**				
Bahrain	98.4	85.2	357.0	311.3	2005
Kuwait	61.9	69.6	281.8	263.4	2008
Oman	81.1	71.8	545.7	333.3	−
Qatar	101.1	84.3	179.8	239.3	2008
S. Arabia	79.2	66.2	540.6	347.6	−
UAE	63.4	64.4	308.9	203.9	−
	**Middle per capita income**				
Algeria	97.7	79.2	278.6	275.0	−
Egypt	107.3	76.1	477.3	384.0	2008
Iraq	120.6	81.7	470.7	376.1	−
Jordan	109.8	89.2	550.4	379.8	2008
Lebanon	151.2	113.2	404.4	262.7	−
Libya	114.3	79.6	458.8	330.1	−
Morocco	90.5	74.5	391.8	319.0	−
Syria	65.7	47.2	471.7	326.2	−
Tunisia	122.6	71.7	267.8	245.4	−
	**Low per capita income**				
Djibouti	95.1	80.4	525.6	452.8	−
Somali	105.3	97.1	570.7	573.4	−
Sudan	78.8	67.6	549.5	545.6	−
Yemen	87.1	80.6	541.8	445.7	−

* Countries with no latest year of data; the estimate of diseases in these countries was based on a combination of country life table, causes of death models, regional cause of death patterns, and WHO programme estimates for some major causes of death. Source: WHO [71].

**Table 5:** Share of some food groups in total available per capita dietary energy consumption (percent) in the Arab countries, according to their income (2003–2005), based on food balance sheet.

Food Group	Low Income	Middle Income	High Income
**Total daily calories intake**	2,000–2,800	2,800–3,000	3,000–3,200
**Cereal (%)**	47–59	34–62	40–48
**Vegetable oils (%)**	11–18	8–18	7–16
**Sugars and sweetness (%)**	10–15	8–15	10–12
**Fruit (%)**	3–4	3–5	2–8
**Vegetables (%)**	4–5	3–4	3–7
**Meat (%)**	4–5	3–8	7–11
**Pulses (%)**	3–5	3–4	2–4
**Milk (%)**	2–18	2–6	4–6

FAO [10].
